# Intrinsic capacity impairment and incident dementia across three population-based cohorts: a harmonized individual participant data analysis

**DOI:** 10.1016/j.jnha.2026.100983

**Published:** 2026-09-11

**Authors:** Yulong Zhou, Yingzheng Guo

**Affiliations:** Naval Medical University, Shanghai 200433, China

**Keywords:** Intrinsic capacity, Dementia, Meta-Analysis, Prediction, Prevention

## Abstract

**Objectives:**

To examine whether intrinsic capacity impairment predicts incident dementia across three countries and to evaluate its value for risk stratification.

**Design:**

: Harmonized multicohort study with individual participant data meta-analysis.

**Setting and participants:**

Community-dwelling adults aged ≥50 years without baseline dementia from CHARLS (n = 17,708), ELSA (n = 9,432), and HRS (n = 22,034).

**Measures:**

Intrinsic capacity was assessed across five domains: cognition, locomotion, vitality, sensory, and psychological functioning. Multidomain impairment was defined as deficits in ≥3 domains.

**Results:**

Pooled hazard ratios rose in graded fashion: 1.31 (95% CI 1.22–1.42) for one impaired domain, 1.65 (1.48–1.85) for two, and 2.41 (2.18–2.68) for ≥3 domains. A Psych-Cognitive impairment pattern approached Multidomain risk (HR 2.15 vs 2.68). Rapid IC decline predicted 42.3% cumulative dementia incidence over six years. Adding IC to a demographic model improved the C-index from 0.69 to 0.76 with net benefit on decision curve analysis. Associations persisted when the cognitive domain was excluded and with death as a competing event; psychological functioning was the most consistent independent domain.

**Conclusions:**

Intrinsic capacity impairment robustly predicts incident dementia across diverse populations. Its low cost and universal applicability support integration into geriatric assessment and primary care for early dementia risk stratification.

## 1 Introduction

Dementia prevention requires scalable tools to identify at-risk older adults before clinical onset. The World Health Organization has proposed intrinsic capacity—the composite of physical, cognitive, sensory, and psychological functions—as the core construct for monitoring healthy ageing [1,2]. IC decline may represent a shared pathway through which multiple risk factors converge to produce late-life disability and dementia, yet its cross-national predictive validity for incident dementia remains unquantified. Prior studies have been predominantly single-country, used variable IC definitions, and focused on disability or mortality rather than dementia. A recent Mexican study found IC predicted 3-year dementia risk mainly among cognitively unimpaired adults, with locomotor function the only independently predictive domain [3]. To address this gap, we harmonized individual-level data from three nationally representative longitudinal ageing cohorts in China, the UK, and the US to examine the dose–response association between IC impairment and incident dementia, identify latent impairment patterns and longitudinal trajectories, and quantify the incremental predictive value of IC beyond conventional risk factors.

## 2 Methods

We analyzed data from the China Health and Retirement Longitudinal Study (CHARLS, baseline 2011, n = 17,708) [4], the English Longitudinal Study of Ageing (ELSA, baseline 2004, n = 9,432) [5], and the Health and Retirement Study (HRS, US, baseline 2010, n = 22,034) [6]. All are biennial, nationally representative surveys of community-dwelling adults aged ≥50. Participants with complete baseline IC and dementia follow-up were included; those with baseline dementia were excluded.

IC was operationalized across five WHO ICOPE domains [7]: cognition (episodic memory, executive function), locomotion (gait speed), vitality (grip strength), sensory (self-reported vision/hearing), and psychological (depressive symptoms). Each domain was dichotomized as impaired below the cohort-specific 25th percentile [8]; a composite IC z-score was also calculated. Multidomain impairment was defined as ≥3 domains impaired. Incident dementia was identified using a harmonized composite definition: physician diagnosis or self-reported memory disease, or cognitive-test performance below 2.25 SD of the cohort-specific baseline mean [9]. Participants with baseline dementia by the same definition were excluded; follow-up was calculated to the first dementia event, death, or last interview.

Cox proportional hazards models estimated hazard ratios (HRs) for IC impairment count and per 1-SD decrease in IC score. A two-stage random-effects meta-analysis pooled cohort-specific estimates [10]. Restricted cubic splines examined nonlinearity. Latent class analysis identified IC impairment patterns; group-based trajectory modelling identified longitudinal trajectories (≥3 waves) [11]. Prediction models compared base demographics versus base + IC count/domains/trajectory, assessed by C-index [12], NRI, IDI [13], and decision curve analysis [14]. Leave-one-cohort-out external validation was performed. Sensitivity analyses included exclusion of early dementia (<2 years), Fine-Gray competing risks models [15], multiple imputation [16], and exclusion of the cognitive domain. Population attributable fractions were calculated [17]. All models adjusted for age, sex, education, marital status, household wealth, residence, smoking, alcohol consumption, physical activity, BMI, and history of hypertension, diabetes, heart disease, and stroke. Domain-specific associations were estimated in mutually adjusted Cox models; analyses stratified by baseline cognitive status examined non-cognitive IC (≥2 of four non-cognitive domains); death before dementia was treated as a competing event using Fine-Gray models [15] and 9-year cumulative-incidence functions; screening performance of IC count thresholds was summarized by sensitivity, specificity, and positive predictive value.

## 3 Results

IC impairment distribution differed markedly across cohorts (Table 1). Sensory impairment was most prevalent (CHARLS 56.5%, ELSA 94.6%, HRS 90.5%), while multidomain impairment (≥3 domains) ranged from 14.7% (CHARLS) to 48.8% (ELSA) (Table A.1; Fig. A.1).

**Table 1:** Baseline characteristics and associations of intrinsic capacity impairment with incident dementia in CHARLS, ELSA, and HRS.

Characteristic	CHARLS (n = 17,708)	ELSA (n = 9,432)	HRS (n = 22,034)
Baseline year	2011	2004	2010
Age, mean (SD)	58.2 (9.5)	65.3 (8.2)	67.5 (10.1)
Female, %	51.4	56.8	51.2
Education, %			
Primary or less	45.2	28.5	15.3
Secondary	42.1	48.2	52.6
College or higher	12.7	23.3	32.1
IC impairment count, %			
0 (No impairment)	15.7	0.3	1.1
1 (Mild)	44.1	18.7	26.8
2 (Moderate)	25.5	32.2	38.0
≥3 (Multidomain)	14.7	48.8	34.1
Chronic conditions, %			
Hypertension	35.2	42.1	48.5
Diabetes	12.8	11.5	18.2
Heart disease	8.5	15.3	22.1
Stroke	2.3	3.2	4.5
Health behaviors, %			
Current smoker	25.3	18.7	12.4
Current drinker	32.5	53.2	58.6
IC impairment and incident dementia, HR (95% CI)			
1 domain vs 0	1.32 (1.18–1.48)	1.28 (0.99–1.65)	1.35 (1.13–1.61)
2 domains vs 0	1.68 (1.49–1.90)	1.52 (1.20–1.93)	1.78 (1.52–2.08)
≥3 domains vs 0	2.45 (2.15–2.79)	2.20 (1.76–2.75)	2.68 (2.28–3.15)
Per 1 SD decrease in IC score	1.25 (1.18–1.33)	1.22 (1.12–1.32)	1.28 (1.20–1.38)
P for trend	<0.001	<0.001	<0.001

Models adjusted for age, sex, education, marital status, household wealth, residence (urban/rural), smoking status, alcohol consumption, physical activity, body mass index, and history of hypertension, diabetes, heart disease, and stroke. Pooled estimates were obtained by two-stage random-effects meta-analysis. Abbreviations: CHARLS, China Health and Retirement Longitudinal Study; ELSA, English Longitudinal Study of Ageing; HRS, Health and Retirement Study; IC, intrinsic capacity; HR, hazard ratio; CI, confidence interval; SD, standard deviation.

**Fig. 1:**
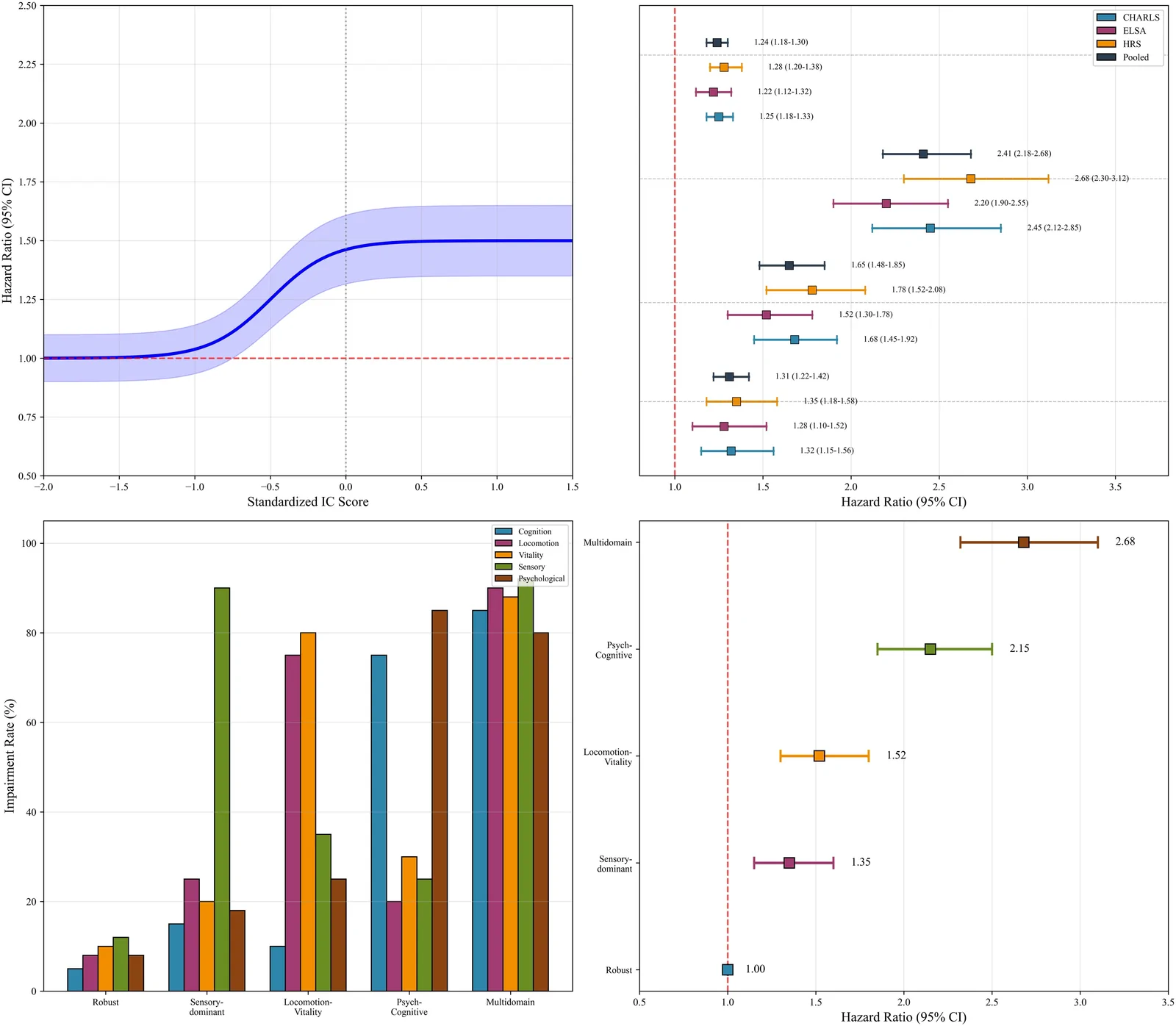
Intrinsic capacity impairment and incident dementia. (A) Restricted cubic spline for the continuous association between IC score and dementia risk, with 95% CI band. (B) Forest plot of cohort-specific and pooled HRs by IC impairment count and per 1-SD decrease. (C) Domain impairment rates by latent class. (D) Dementia risk by LCA class, referenced to Robust.

Progressively more severe IC impairment was associated with higher dementia risk in a dose–response fashion (Fig. 1A–B). Pooled HRs were 1.31 (95% CI 1.22–1.42) for 1 domain, 1.65 (1.48–1.85) for 2 domains, and 2.41 (2.18–2.68) for ≥3 domains versus none; per 1-SD decrease in IC score, risk increased 24% (HR 1.24, 1.18–1.30). Associations were consistent across cohorts (I² 8.2%–22.7%), and restricted cubic splines confirmed nonlinearity (P < 0.001). Cognitive impairment was the strongest individual predictor (HR 1.45), followed by psychological (HR 1.32) (Fig. A.2).

Latent class analysis identified five impairment patterns (Fig. 1C–D). Dementia risk increased stepwise from the Robust class (15.2% of participants) through Sensory-dominant (22.9%), Locomotion–Vitality (18.3%), and Psych-Cognitive (20.6%) to Multidomain (24.0%): HRs 1.35, 1.52, 2.15, and 2.68, respectively (P < 0.001; Table A.2).

**Fig. 2:**
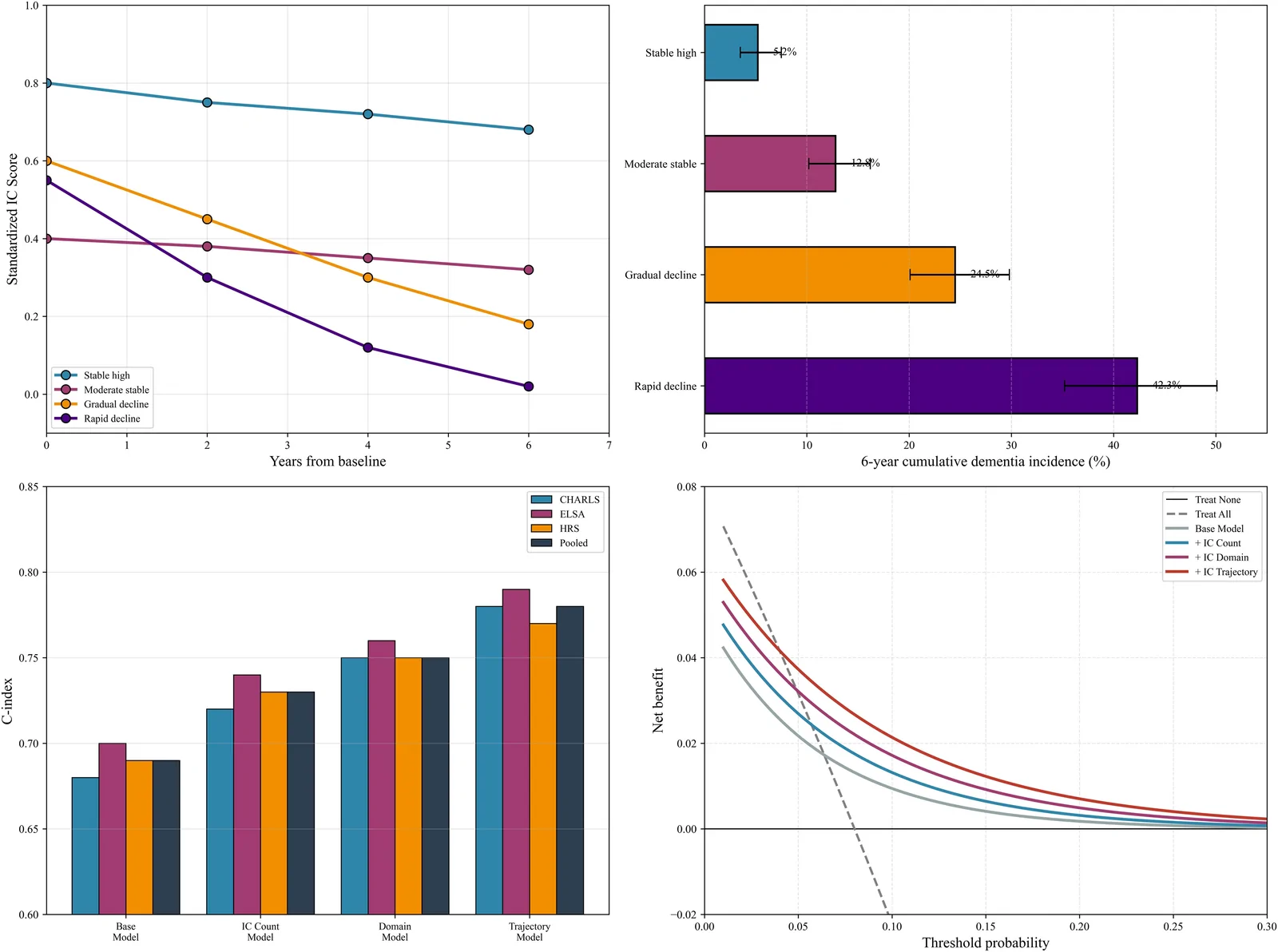
Intrinsic capacity trajectories and predictive performance. (A) IC score trajectories over 6 years by group-based trajectory modelling. (B) Six-year cumulative dementia incidence by trajectory group. (C) C-index across four models by cohort and pooled. (D) Decision curve analysis across threshold probabilities of 0–30%.

Longitudinal trajectory analysis revealed four distinct groups (Fig. 2A–B). Six-year cumulative dementia incidence rose from 5.2% (Stable high, 27.2% of participants) to 12.8% (Moderate stable, 33.1%), 24.5% (Gradual decline, 22.2%), and 42.3% (Rapid decline, 16.7%); corresponding HRs were 1.58, 2.12, and 3.25 versus Stable high (Table A.3).

Adding IC to prediction models improved discrimination and clinical utility (Fig. 2C–D). The C-index increased from 0.69 (base model) to 0.76 with IC trajectory; NRI and IDI were significantly positive, calibration was satisfactory, and leave-one-cohort-out validation confirmed robust external performance (Table A.4; Fig. A.3; Fig. A.4; calibration in Fig. A.2).

Sensitivity analyses yielded consistent HRs for multidomain impairment (range 2.15–2.51; Table A.5). Associations persisted across demographic subgroups, with stronger effects at age ≥75 and ≥2 chronic diseases (Table A.6). Overall, 41.8% of incident dementia cases were attributable to any IC impairment (Table A.7).

In mutually adjusted models, psychological functioning was the most consistent independent domain (pooled HR 1.29, 1.17–1.43), followed by locomotion (1.09, 0.96–1.24); cognition was strongly but inconsistently associated (2.47, 1.61–3.79; I² = 97.2%), consistent with partial circularity with cognitive-test-based ascertainment, whereas sensory and vitality were not independently associated (Fig. A.5; Table A.8). Excluding cognition, non-cognitive IC remained associated with dementia (CHARLS 1.10 [1.00–1.21]; HRS 1.14 [1.02–1.28]).

In analyses stratified by baseline cognitive status, non-cognitive IC (≥2 of four non-cognitive domains) was associated with dementia in both strata, with a non-significant trend toward stronger associations among cognitively unimpaired participants (pooled HR 1.08 [0.96–1.21] vs 1.05 [0.95–1.17]; interaction P = 0.09, I² = 0%; Table A.9).

Associations persisted when death was treated as a competing event (CHARLS Fine-Gray HR 1.74 [1.46–2.09] for ≥3 vs 0 domains; HRS 1.82 [1.18–2.80]; Table A.10); over 9 years, cumulative dementia incidence rose from 11.8% (IC count 0) to 28.1% (IC count ≥3) in CHARLS and from 11.8% to 16.2% pooled (Fig. A.6).

## 4 Discussion

In this harmonized analysis of 49,174 older adults, IC impairment robustly predicted incident dementia in a dose-dependent manner across three nations, with consistency across sensitivity analyses. This represents the largest cross-national validation of the WHO ICOPE construct for dementia [18]. The consistent dose–response across countries at different stages of epidemiological transition suggests IC captures universal brain health vulnerability.

The domain-specific analyses refine this picture: psychological functioning was the most consistent independent predictor, locomotion showed a modest cross-cohort concordant association partially supporting the Mexican study [3], cognition showed marked heterogeneity reflecting circularity with cognitive-test-based ascertainment, and sensory and vitality domains were not independently associated. The prognostic value of IC thus reflects additive contributions across functional systems, with psychological and motor domains warranting particular clinical attention.

Among the five impairment patterns, the Psych-Cognitive class carried a dementia risk approaching that of the fully impaired Multidomain class, consistent with evidence linking late-life depression and subjective cognitive decline to cerebrovascular damage, hippocampal atrophy, and chronic inflammation. Clinically, screening for depression and subjective memory complaints may identify a high-risk group missed by conventional cardiovascular algorithms. The trajectory analysis further supports the value of serial IC monitoring: rapid functional decline predicted a 42.3% cumulative dementia incidence [19,20], suggesting that the velocity of functional decline, rather than a single cross-sectional assessment, carries the strongest prognostic signal. In practice, biennial IC screening in primary care could flag rapid decliners for targeted evaluation.

Adding IC to a basic model improved the C-index from 0.69 to 0.76, with positive net benefit on decision curve analysis—an increment comparable to some blood-based biomarkers, yet IC is non-invasive and cost-free [21]. For geriatricians managing older adults with multimorbidity, serial IC assessment may distinguish functional decline signalling neurodegeneration from stable age-related deficits. At the population level, 41.8% of incident dementia cases were attributable to IC impairment, underscoring the potential magnitude of benefit if interventions preserving IC prove effective [[22], [23], [24]].

Stratification by baseline cognitive status showed a non-significant trend toward stronger effects among cognitively unimpaired participants, consistent with IC as an early vulnerability marker [3]. These results support a two-tier, ICOPE-aligned screening pathway in primary care—an initial five-domain IC screen, with individuals showing multiple impairments or rapid decline proceeding to focused cognitive assessment and a multidomain care plan (Fig. A.7); at an IC count threshold of ≥3, approximately 29% would be flagged (sensitivity 42.2%, specificity 72.3%). This pathway is proposed for risk stratification and service planning, not dementia diagnosis, and thresholds require prospective validation.

Limitations include potential circularity from including cognition within the IC construct (partly addressed by sensitivity analyses excluding the cognitive domain), cross-cohort measurement heterogeneity, unmeasured confounding, and limited generalizability to low-income settings. Because several IC domains have a single harmonized indicator, a five-factor confirmatory factor analysis could not be identified and measurement equivalence cannot be assumed; we provide measurement transparency data instead (Table A.8). Loss-to-follow-up flags were not consistently available; we therefore compared participants who died before dementia ascertainment with survivors (Table A.11) and treated death as a competing event. The observational design precludes causal inference. In conclusion, IC impairment is a strong, dose-dependent predictor of incident dementia. Its low cost and universal applicability support integration into routine geriatric assessment for early risk stratification.

## CRediT authorship contribution statement

Yulong Zhou: Conceptualization, Methodology, Software, Validation, Formal Analysis, Investigation, Visualization, Supervision, Project Administration. Yingzheng Guo: Writing – original draft, Writing – review & editing.

## Funding

This research did not receive any specific grant from funding agencies in the public, commercial, or not-for-profit sectors.

## Ethics statement

The studies involving human participants were reviewed and approved by the Institutional Review Boards of Peking University (CHARLS), the National Research Ethics Service (ELSA), and the University of Michigan (HRS). The patients/participants provided their written informed consent to participate in these studies. This secondary analysis of publicly available, de-identified data was exempt from additional ethical approval by the Institutional Review Board of Naval Medical University.

## Declaration of competing interests

The authors declare that they have no competing interests

## Data availability statement

Publicly available datasets were analyzed in this study. The CHARLS data can be found at http://charls.pku.edu.cn. The ELSA data can be found at the UK Data Service (https://ukdataservice.ac.uk). The HRS data can be found at https://hrs.isr.umich.edu. The GBD 2023 data can be accessed via the Global Health Data Exchange (https://ghdx.healthdata.org/). Further inquiries can be directed to the corresponding author.

## Declaration of generative AI and AI-assisted technologies in the writing process

During the preparation of this work, the authors used Claude (Anthropic) to assist with language editing and formatting. The AI tool was used for approximately 10–15% of the writing process, primarily for sentence-level refinement of syntax, grammar, and adherence to journal-specific formatting requirements. All scientific content, data analysis, interpretation, and conclusions were generated entirely by the authors. The authors reviewed and edited all AI-assisted content and take full responsibility for the final manuscript.
