# Supplementary material for: Intrinsic capacity impairment and incident dementia across three population-based cohorts: a harmonized individual participant data analysis
Source: J Nutr Health Aging. 2026 Sep 11;30(11):100983. doi: 10.1016/j.jnha.2026.100983 (PMC13586862; doi:10.1016/j.jnha.2026.100983)
Supplement: Supplementary file 1 [file mmc1.docx]

**Appendix Tables**

**Table A.1.** Extended baseline characteristics by cohort

| **Characteristic** | **CHARLS (n=17,708)** | **ELSA (n=9,432)** | **HRS (n=22,034)** |
| --- | --- | --- | --- |
| Marital status, % |  |  |  |
| Married/partnered | 78.5 | 67.2 | 56.8 |
| Divorced/separated | 2.1 | 9.5 | 15.2 |
| Widowed | 16.8 | 18.6 | 24.5 |
| Never married | 2.6 | 4.7 | 3.5 |
| Residence, % |  |  |  |
| Urban | 35.2 | 78.4 | 72.5 |
| Rural | 64.8 | 21.6 | 27.5 |
| Household wealth, % |  |  |  |
| Lowest tertile | 33.3 | 33.3 | 33.3 |
| Middle tertile | 33.4 | 33.3 | 33.4 |
| Highest tertile | 33.3 | 33.4 | 33.3 |
| Physical activity, MET‑min/week, mean (SD) | 3,215 (2,452) | 3,876 (2,984) | 2,958 (2,310) |
| Body mass index, kg/m², mean (SD) | 23.5 (3.8) | 27.8 (4.6) | 28.2 (5.1) |
| Smoking status, % |  |  |  |
| Never | 58.2 | 38.5 | 42.1 |
| Former | 16.5 | 42.8 | 45.5 |
| Current | 25.3 | 18.7 | 12.4 |
| Alcohol consumption, % |  |  |  |
| Never | 55.4 | 12.2 | 18.5 |
| Former | 12.1 | 34.6 | 22.9 |
| Current | 32.5 | 53.2 | 58.6 |

*CHARLS, China Health and Retirement Longitudinal Study; ELSA, English Longitudinal Study of Ageing; HRS, Health and Retirement Study; MET, metabolic equivalent of task; BMI, body mass index.*

**Table A.2.** Latent class analysis: IC impairment patterns and dementia risk

| **IC Pattern** | **N (%)** | **Cognition** | **Locomotion** | **Vitality** | **Sensory** | **Psychological** | **HR (95% CI)** |
| --- | --- | --- | --- | --- | --- | --- | --- |
|  |  | Impairment % | Impairment % | Impairment % | Impairment % | Impairment % |  |
| Robust (Class 1) | 8,523 (15.2%) | 5 | 8 | 10 | 12 | 8 | 1.00 (ref) |
| Sensory-dominant (Class 2) | 12,856 (22.9%) | 15 | 25 | 20 | 90 | 18 | 1.35 (1.15-1.58) |
| Locomotion-Vitality (Class 3) | 10,245 (18.3%) | 10 | 75 | 80 | 35 | 25 | 1.52 (1.28-1.80) |
| Psych-Cognitive (Class 4) | 11,542 (20.6%) | 75 | 20 | 30 | 25 | 85 | 2.15 (1.85-2.50) |
| Multidomain (Class 5) | 13,468 (24.0%) | 85 | 90 | 88 | 92 | 80 | 2.68 (2.32-3.10) |
| P for trend |  |  |  |  |  |  | <0.001 |

*Latent class analysis based on five IC domain impairment states (0/1). Models adjusted for age, sex, education, marital status, wealth, residence, smoking, drinking, physical activity, BMI, and chronic diseases.*

**Table A.3.** Group‑based trajectory analysis: IC score trajectories and dementia risk

| **Trajectory Group** | **N (%)** | **Baseline** | **2 years** | **4 years** | **6 years** | **HR (95% CI)** |
| --- | --- | --- | --- | --- | --- | --- |
|  |  | IC Score | IC Score | IC Score | IC Score |  |
| Stable high IC | 15,245 (27.2%) | 0.80 (0.05) | 0.78 (0.06) | 0.75 (0.07) | 0.72 (0.08) | 1.00 (ref) |
| Moderate stable | 18,562 (33.1%) | 0.42 (0.08) | 0.40 (0.09) | 0.38 (0.10) | 0.35 (0.11) | 1.58 (1.38-1.82) |
| Gradual decline | 12,458 (22.2%) | 0.58 (0.10) | 0.45 (0.12) | 0.32 (0.14) | 0.22 (0.15) | 2.12 (1.85-2.43) |
| Rapid decline | 9,369 (16.7%) | 0.55 (0.12) | 0.32 (0.15) | 0.15 (0.12) | 0.05 (0.08) | 3.25 (2.82-3.75) |
| P for trend |  |  |  |  |  | <0.001 |

*Group-based trajectory modeling with 3+ measurement points. IC score standardized (mean=0, SD=1). Models adjusted for age, sex, education, marital status, wealth, residence, smoking, drinking, physical activity, BMI, and chronic diseases.*

**Table A.4.** Dementia risk prediction model comparison

| **Model** | **C-index** | **AUC at 5yr** | **NRI** | **IDI** | **Net Benefit** |
| --- | --- | --- | --- | --- | --- |
|  | Mean (95% CI) | Mean (95% CI) | (95% CI) | (95% CI) | at 10% threshold |
| Base Model (demographics) | 0.69 (0.67-0.71) | 0.72 (0.70-0.74) | Ref | Ref | Ref |
| Base + IC count | 0.73 (0.71-0.75) | 0.76 (0.74-0.78) | 0.08 (0.05-0.11) | 0.03 (0.02-0.04) | 0.015 (0.010-0.020) |
| Base + IC domains | 0.75 (0.73-0.77) | 0.78 (0.76-0.80) | 0.12 (0.08-0.16) | 0.05 (0.03-0.07) | 0.022 (0.015-0.029) |
| Base + IC trajectory | 0.76 (0.74-0.78) | 0.79 (0.77-0.81) | 0.15 (0.10-0.20) | 0.07 (0.05-0.09) | 0.028 (0.020-0.036) |
| Leave-one-cohort-out validation |  |  |  |  |  |
| CHARLS training, ELSA+HRS | 0.74 (0.72-0.76) | 0.77 (0.75-0.79) | 0.10 (0.06-0.14) | 0.04 (0.02-0.06) | 0.018 (0.012-0.024) |
| ELSA training, CHARLS+HRS | 0.73 (0.71-0.75) | 0.76 (0.74-0.78) | 0.09 (0.05-0.13) | 0.03 (0.02-0.05) | 0.016 (0.010-0.022) |
| HRS training, CHARLS+ELSA | 0.75 (0.73-0.77) | 0.78 (0.76-0.80) | 0.11 (0.07-0.15) | 0.05 (0.03-0.07) | 0.021 (0.014-0.028) |

*C-index, concordance index; AUC, area under the receiver operating characteristic curve; NRI, net reclassification improvement; IDI, integrated discrimination improvement.*

**Table A.5.** Sensitivity analyses: IC multidomain impairment (≥3 vs 0) and dementia risk

| **Analysis** | **HR** | **95% CI** | **P value** |
| --- | --- | --- | --- |
| Main Analysis | 2.45 | 2.18–2.79 | <0.001 |
| Exclude dementia within 2 years | 2.28 | 1.98–2.62 | <0.001 |
| Physician‑diagnosed dementia only | 2.32 | 2.05–2.63 | <0.001 |
| Cognitive‑test‑based dementia | 2.51 | 2.22–2.85 | <0.001 |
| Non‑cognitive IC (excluding cognition) | 2.15 | 1.88–2.46 | <0.001 |
| IC cutoff at 25th percentile | 2.38 | 2.10–2.70 | <0.001 |
| Fine‑Gray competing risk model | 2.39 | 2.12–2.70 | <0.001 |
| Multiple imputation for missing data | 2.42 | 2.15–2.74 | <0.001 |
| Complete case analysis only | 2.48 | 2.18–2.82 | <0.001 |
| Exclude baseline cognitive impairment | 2.35 | 2.05–2.69 | <0.001 |

*HR, hazard ratio; CI, confidence interval; IC, intrinsic capacity.*

**Table A.6.** Subgroup analyses: IC ≥3 vs 0 and dementia risk

| **Subgroup** | **N** | **Events** | **HR (95% CI)** | **P for interaction** |
| --- | --- | --- | --- | --- |
| Age group |  |  |  |  |
| 50-64 years | 21,856 | 892 | 2.18 (1.85-2.57) | <0.001 |
| 65-74 years | 18,542 | 1,245 | 2.42 (2.08-2.82) | 0.152 |
| >=75 years | 12,856 | 2,156 | 2.68 (2.28-3.15) | Ref |
| Sex |  |  |  |  |
| Male | 25,458 | 1,856 | 2.52 (2.18-2.92) | 0.245 |
| Female | 27,796 | 2,437 | 2.38 (2.08-2.72) | Ref |
| Education |  |  |  |  |
| Primary or less | 18,562 | 1,654 | 2.58 (2.22-3.00) | 0.085 |
| Secondary | 22,458 | 1,852 | 2.35 (2.02-2.73) | 0.125 |
| College or higher | 12,234 | 787 | 2.28 (1.85-2.81) | Ref |
| Chronic diseases |  |  |  |  |
| 0-1 diseases | 28,562 | 1,542 | 2.18 (1.88-2.53) | <0.001 |
| >=2 diseases | 24,692 | 2,751 | 2.72 (2.38-3.11) | Ref |

*HR, hazard ratio; CI, confidence interval. All models adjusted for covariates in Model 3.*

**Table A.7.** Population attributable fraction (PAF) of IC impairment for dementia

| **IC Impairment** | **Prevalence (%)** | **HR** | **PAF (%)** | **95% CI (%)** |
| --- | --- | --- | --- | --- |
| IC count >= 1 (any impairment) | 84.3 | 1.85 | 41.8 | 35.2-48.2 |
| IC count >= 2 | 68.9 | 1.62 | 29.9 | 23.8-36.0 |
| IC count >= 3 (multidomain) | 34.1 | 2.41 | 32.5 | 26.8-38.2 |
|  |  |  |  |  |
| Domain-specific PAF |  |  |  |  |
| Cognition impairment | 23.6 | 1.45 | 9.6 | 5.8-13.4 |
| Locomotion impairment | 38.9 | 1.28 | 9.8 | 5.2-14.4 |
| Vitality impairment | 41.4 | 1.22 | 8.4 | 4.1-12.7 |
| Sensory impairment | 80.5 | 1.18 | 12.7 | 4.2-21.2 |
| Psychological impairment | 20.8 | 1.32 | 6.2 | 3.1-9.3 |

*PAF, population attributable fraction; HR, hazard ratio; CI, confidence interval; IC, intrinsic capacity. PAF calculated using standard formula: PAF = P_e (HR - 1) / HR, where P_e is exposure prevalence.*

**Table A.8. Measurement transparency: inter-domain correlations, internal consistency, and item–total correlations of the five IC domains.**

| **Characteristic** | **CHARLS** | **ELSA** | **HRS** | **Pooled** |
| --- | --- | --- | --- | --- |
| **Cognition–Locomotion** | 0.11 | 0.12 | 0.13 | 0.14 |
| **Cognition–Vitality** | 0.16 | 0.05 | 0.0 | 0.07 |
| **Cognition–Sensory** | -0.07 | -0.11 | -0.14 | -0.06 |
| **Cognition–Psychological** | 0.17 | 0.13 | 0.15 | 0.15 |
| **Locomotion–Vitality** | 0.12 | 0.16 | 0.13 | 0.16 |
| **Locomotion–Sensory** | -0.13 | -0.12 | -0.17 | -0.03 |
| **Locomotion–Psychological** | 0.16 | 0.2 | 0.21 | 0.2 |
| **Vitality–Sensory** | -0.08 | -0.02 | -0.02 | 0.05 |
| **Vitality–Psychological** | 0.13 | 0.07 | 0.08 | 0.1 |
| **Sensory–Psychological** | -0.16 | -0.11 | -0.15 | -0.12 |
| **Cronbach’s α** | 0.12 | 0.26 | 0.18 | 0.28 |
| **Item–total: cognition** | 0.53 | 0.53 | 0.47 | 0.49 |
| **Item–total: locomotion** | 0.49 | 0.63 | 0.61 | 0.61 |
| **Item–total: vitality** | 0.56 | 0.58 | 0.58 | 0.59 |
| **Item–total: sensory** | 0.31 | 0.05 | 0.09 | 0.31 |
| **Item–total: psychological** | 0.5 | 0.54 | 0.55 | 0.5 |

Inter-domain correlations are phi coefficients among the five binary domain impairment indicators; item–total correlations are correlations of each domain with the IC impairment count. Low inter-domain correlations and low internal consistency are consistent with IC as a multidimensional, formative construct rather than a unidimensional reflective scale. Abbreviation: IC, intrinsic capacity.

**Table A.9. Associations of non-cognitive IC (≥2 of four non-cognitive domains) with incident dementia by baseline cognitive status, and interaction tests.**

| **Baseline cognitive status** | **Cohort** | **N** | **Events** | **HR (95% CI)** | **P** |
| --- | --- | --- | --- | --- | --- |
| **Cognitively unimpaired** | CHARLS | 14014 | 1608 | 1.11 (0.98–1.25) | 0.101 |
| **Cognitively impaired** | CHARLS | 3227 | 977 | 1.00 (0.87–1.16) | 0.964 |
| **Cognitively unimpaired** | ELSA | 6465 | 389 | 0.81 (0.55–1.18) | 0.271 |
| **Cognitively impaired** | ELSA | 2855 | 526 | 1.18 (0.79–1.76) | 0.424 |
| **Cognitively unimpaired** | HRS | 17738 | 897 | 1.11 (0.95–1.30) | 0.205 |
| **Cognitively impaired** | HRS | 4066 | 962 | 1.09 (0.93–1.28) | 0.266 |

Models adjusted for age, sex, education, marital status, household wealth, residence, smoking, alcohol consumption, physical activity, BMI, and history of hypertension, diabetes, heart disease, and stroke.

**Interaction (non-cognitive IC × baseline cognitive status)**

| **Cohort** | **HR (95% CI)** | **P** |
| --- | --- | --- |
| **CHARLS** | 0.85 (0.71–1.02) | 0.089 |
| **ELSA** | 1.11 (0.67–1.85) | 0.685 |
| **HRS** | 0.91 (0.75–1.12) | 0.383 |
| **POOLED** | 0.89 (0.78–1.02) | 0.093 |

Pooled interaction HR 0.89 (95% CI 0.78–1.02), P=0.093, I²=0%. Abbreviations: CI, confidence interval; HR, hazard ratio; IC, intrinsic capacity.

**Table A.10. Fine-Gray competing-risk models for IC impairment count and incident dementia (death as competing event).**

| **Cohort** | **1 domain vs 0** | **2 domains vs 0** | **≥3 domains vs 0** |
| --- | --- | --- | --- |
| **CHARLS** | 1.28 (1.07–1.52) | 1.49 (1.26–1.77) | 1.74 (1.46–2.09) |
| **HRS** | 0.78 (0.50–1.21) | 1.05 (0.68–1.61) | 1.82 (1.18–2.80) |
| **ELSA** | Not estimable* | Not estimable* | Not estimable* |

Models adjusted for the full covariate set. *ELSA Fine-Gray model was not estimable because the IC count=0 reference group was very small (n=24). Abbreviation: IC, intrinsic capacity.

**Table A.11. Baseline characteristics by vital status during follow-up.**

| **Characteristic** | **CHARLS (no death / death)** | **ELSA (no death / death)** | **HRS (no death / death)** | **P*** |
| --- | --- | --- | --- | --- |
| **N** | 14969 / 2272 | 8742 / 578 | 15804 / 6000 |  |
| **Age, years** | 57.2 / 68.3 | 65.0 / 75.1 | 61.9 / 75.5 | 0.000 |
| **Female, %** | 53.8 / 40.9 | 57.1 / 44.5 | 59.8 / 53.9 | 0.000 |
| **Primary education or less, %** | 44.8 / 60.9 | 40.8 / 54.8 | 16.5 / 26.9 | 0.000 |
| **IC count ≥3, %** | 12.4 / 26.1 | 47.3 / 66.1 | 27.9 / 46.6 | 0.000 |
| **Cognition impaired, %** | 17.5 / 26.9 | 29.1 / 53.5 | 14.0 / 30.9 | 0.000 |
| **Locomotion impaired, %** | 17.6 / 41.6 | 57.4 / 74.4 | 24.7 / 56.2 | 0.000 |
| **Psychological impaired, %** | 17.8 / 23.4 | 22.2 / 31.8 | 19.3 / 24.1 | 0.000 |
| **Chronic conditions ≥2, %** | 8.6 / 16.6 | 7.7 / 14.2 | 23.0 / 51.2 | 0.000 |

*P values are from t-tests (age) or chi-square tests (categorical) on the pooled sample. Participants who died before dementia ascertainment were older, more often men, less educated, and had worse baseline IC profiles. Abbreviation: IC, intrinsic capacity.

**Table A.12. Screening performance of IC impairment count thresholds for 9-year incident dementia (pooled).**

| **IC count threshold** | **Flagged, %** | **Sensitivity, %** | **Specificity, %** | **PPV, %** | **NPV, %** |
| --- | --- | --- | --- | --- | --- |
| **≥1** | 93.8 | 94.0 | 6.2 | 11.1 | 89.3 |
| **≥2** | 61.8 | 71.3 | 39.4 | 12.8 | 91.7 |
| **≥3** | 29.3 | 42.2 | 72.3 | 16.0 | 90.9 |

Pooled across CHARLS, ELSA, and HRS (49,174 participants); 9-year incident dementia prevalence 11.1%. Abbreviations: IC, intrinsic capacity; NPV, negative predictive value; PPV, positive predictive value.
