# Supplementary material for: Intrinsic capacity impairment and incident dementia across three population-based cohorts: a harmonized individual participant data analysis
Source: J Nutr Health Aging. 2026 Sep 11;30(11):100983. doi: 10.1016/j.jnha.2026.100983 (PMC13586862; doi:10.1016/j.jnha.2026.100983)
Supplement: Supplementary file 2 [file mmc2.docx]

**Appendix Figures**

**Fig. A.1.** Intrinsic capacity impairment landscape and divergent dementia epidemics across China, the UK, and the US.

*Panel A shows the prevalence of impairment in each IC domain in CHARLS, ELSA, and HRS. Panel B displays the distribution of the number of impaired IC domains by cohort. Panel C presents age-standardised dementia prevalence rates from 1990 to 2023 in the three countries; shaded areas indicate 95% uncertainty intervals from the Global Burden of Disease Study 2023. Panel D shows prevalence in 2023 with 95% uncertainty intervals. Abbreviations: CHARLS, China Health and Retirement Longitudinal Study; ELSA, English Longitudinal Study of Ageing; HRS, Health and Retirement Study; IC, intrinsic capacity.*

**Fig. A.2.** Robustness, specificity, and calibration of the association between intrinsic capacity impairment and dementia.

*Panel A displays hazard ratios and 95% confidence intervals from sensitivity analyses for multidomain IC impairment (≥3 vs 0) and incident dementia. Panel B shows cohort-specific pooled hazard ratios for each individual IC domain. Panel C presents subgroup analyses by age, sex, education, and number of chronic diseases. Panel D shows the calibration plot for 6-year dementia risk, comparing predicted and observed probabilities for the Base Model and the IC Count Model; the diagonal line indicates perfect calibration. Abbreviations: CI, confidence interval; HR, hazard ratio; IC, intrinsic capacity.*

**Fig. A.3.**Net reclassification improvement and integrated discrimination improvement of IC-enhanced models compared with the Base Model.

*Bars represent point estimates with 95% confidence intervals. The Base Model included demographics only. IC Count, Domain, and Trajectory models additionally incorporated the corresponding IC measures.*

**Fig. A.4.** Leave-one-cohort-out external validation of the IC-enhanced prediction model: C-index with 95% confidence intervals.

*Each point represents the C‑index with 95% confidence interval when training on one cohort (e.g., CHARLS) and testing on the remaining two cohorts (e.g., ELSA and HRS pooled). The IC Count Model was used for all external validations.*

**Fig. A.5.** Mutually adjusted, fully adjusted domain-specific hazard ratios for incident dementia by cohort and pooled.

*Hazard ratios are from Cox proportional hazards models including all five IC domains simultaneously, adjusted for age, sex, education, marital status, household wealth, residence, smoking, alcohol consumption, physical activity, BMI, and history of hypertension, diabetes, heart disease, and stroke. Pooled estimates were obtained by two-stage random-effects meta-analysis; I² values: cognition 97.2%, locomotion 66.4%, vitality 72.2%, sensory 20.8%, psychological 43.4%. Abbreviations: CI, confidence interval; HR, hazard ratio; IC, intrinsic capacity.*

**Fig. A.6.** Nine-year cumulative dementia incidence with death as a competing event, by IC impairment count (pooled).

*Cumulative incidence functions treat death before dementia as a competing event over the common 9-year follow-up window across CHARLS, ELSA, and HRS. Abbreviations: IC, intrinsic capacity.*

**Fig. A.7.** Two-tier, IC-informed primary-care risk-stratification pathway.

*Tier 1 comprises five-domain IC screening in primary care; individuals with ≥2 impaired domains or rapid functional decline proceed to Tier 2 (focused cognitive assessment and a multidomain care plan). Screening metrics were derived from the pooled 49,174-participant cohort over 9 years (IC count ≥3: 29.3% flagged, sensitivity 42.2%, specificity 72.3%, positive predictive value 16.0%; IC count ≥2: 61.8% flagged, sensitivity 71.3%, specificity 39.4%). The pathway is proposed for risk stratification and service planning, not for dementia diagnosis, and implementation thresholds require prospective validation. Abbreviations: IC, intrinsic capacity; PPV, positive predictive value.*
